# Humidification during laparoscopic surgery: overview of the clinical benefits of using humidified gas during laparoscopic surgery

**DOI:** 10.1007/s00404-015-3717-y

**Published:** 2015-04-25

**Authors:** Maria Mercedes Binda

**Affiliations:** Institut de Recherche Expérimentale et Clinique (IREC), Pôle de Gynécologie, Avenue Mounier 52, bte B1.52.02, 1200 Brussels, Belgium

**Keywords:** Laparoscopy, Pneumoperitoneum, Humidity, Body temperature, Pain, Post-operative adhesions

## Abstract

**Purpose:**

The peritoneum is the serous membrane that covers the abdominal cavity and most of the intra-abdominal organs. It is a very delicate layer highly susceptible to damage and it is not designed to cope with variable conditions such as the dry and cold carbon dioxide (CO_2_) during laparoscopic surgery. The aim of this review was to evaluate the effects caused by insufflating dry and cold gas into the abdominal cavity after laparoscopic surgery.

**Methods:**

A literature search using the Pubmed was carried out. Articles identified focused on the key issues of laparoscopy, peritoneum, morphology, pneumoperitoneum, humidity, body temperature, pain, recovery time, post-operative adhesions and lens fogging.

**Results:**

Insufflating dry and cold CO_2_ into the abdomen causes peritoneal damage, post-operative pain, hypothermia and post-operative adhesions. Using humidified and warm gas prevents pain after surgery. With regard to hypothermia due to desiccation, it can be fully prevented using humidified and warm gas. Results relating to the patient recovery are still controversial.

**Conclusions:**

The use of humidified and warm insufflation gas offers a significant clinical benefit to the patient, creating a more physiologic peritoneal environment and reducing the post-operative pain and hypothermia. In animal models, although humidified and warm gas reduces post-operative adhesions, humidified gas at 32 °C reduced them even more. It is clear that humidified gas should be used during laparoscopic surgery; however, a question remains unanswered: to achieve even greater clinical benefit to the patient, at what temperature should the humidified gas be when insufflated into the abdomen? More clinical trials should be performed to resolve this query.

## Basics of the physiology of the peritoneum

The peritoneum is the serous membrane that forms the lining of the abdominal cavity and it covers most of the intra-abdominal organs. It is composed of a single layer of mesothelium, generally 2.5–3 µm thick, supported by a thin layer of connective tissue [1]. With a surface area of some 14,000 cm^2^ in adults [2], almost equal to that of the skin, this membrane may be the largest organ in humans. Its function is to diminish the friction among abdominal viscera, enabling their free movement. It also walls off infection and serves as a reservoir of fat, especially in the omentum. It contains two distinct layers of collagen, and it is one of the most richly vascularised of all organs. The membrane comprises very large amounts of mucopolysaccharides or glycosaminoglycans and just beneath its surface there is an elastin layer that gives the peritoneum mobility. The surface lining of the peritoneum consists of highly differentiated mesothelial cells [3].

Mesothelial cells are predominantly flattened, squamous-like, approximately 25 μm in diameter, with the cytoplasm raised over a central round or oval nucleus [4]. Long microvilli are projected from the apical surface of the mesothelial cells [1]. They have well-developed cell-to-cell junctional complexes including tight junctions, adherent junctions, gap junctions and desmosomes. Tight junctions, in particular, are crucial for the development of cell surface polarity and the establishment and maintenance of a semi-permeable diffusion barrier [4]. They secrete glycosaminoglycans, proteoglycans and phospholipids to provide a slippery, non-adhesive glycocalyx that protects the serosal surface from abrasion, infection and tumour dissemination. In addition, mesothelial cells can synthesise cytokines, chemokines, growth factors and matrix components that regulate inflammation, initiate cell proliferation, differentiation and migration, and mediate tissue repair [5]. Providing scaffolding for the mesothelial cells are connective tissue proteins, and abundant vascular channels deliver oxygen and other nutrients to them. Interspersed among the connective tissue, there are extremely poorly differentiated and epithelioid-like cells similar to fibroblasts. These cells can undergo a variety of differentiation changes after exposure to injury or other types of stimuli, perhaps becoming mesothelial cells during peritoneal repair [3].

In summary, the peritoneal surface has a very important function in the abdominal cavity, i.e. to diminish the friction, wall off infection and to enable the secretion of cytokines. However, it is a very delicate layer and, therefore, highly susceptible to being damaged.

## Laparoscopic surgery

Laparoscopy induces less direct trauma because of gentle tissue handling, meticulous haemostasis, constant irrigation, the use of microsurgical instruments and the smaller operative field. This procedure has been associated with less post-operative pain, less systemic immunological depression, less wound infection, fewer complications, faster bowel recovery, shorter hospital stays and earlier return to normal activities; however, the operating times can be longer in comparison to those in open surgery [6–11].

Typically, during laparoscopic surgery, the abdominopelvic cavity is first inflated with a gas to provide a space for viewing the surgical site and manipulating instruments. CO_2_ is used almost universally as the insufflation agent to create this space called the laparoscopic pneumoperitoneum. CO_2_ is the most common gas used for insufflation because of safety and supply reasons. First, it is non-combustible, eliminating the risk of fire when electro-surgical instruments are used, and second, it is cheap and highly soluble in water [12]. Solubility is important as any gas trapped in the body following surgery must be removed. CO_2_ dissolves into the serous fluid then migrates into the bloodstream where it travels to the lungs and is breathed out; therefore, CO_2_ can easily be removed from the body without any major effect on the body’s metabolism. This high solubility in water reduces the risk of gas embolism impairing cardiac function.

The peritoneum is not designed to cope with variable conditions such as the introduction of dry and cold gas. Any change in the environment has an impact: the larger the deviation from physiologic intra-abdominal conditions, the larger the effect. Thus, the type of gas insufflated in the abdominal cavity (CO_2_ or other gases), the nature of the gas (its temperature and humidity), the pressure and the extent of exposure to this gas (combination of time and volume of the gas) are factors that cause tissue damage.

Currently, dry CO_2_ gas at room temperature is used for insufflation. However, significant evidence suggests the use of humid and warm gas may reduce at least two of the major morbidities associated with laparoscopic surgery: post-operative pain and hypothermia [13, 14]. Humidifying insufflation gas provides a more physiologically normal pneumoperitoneum. This is a logical progression towards minimising trauma in line with the philosophy of laparoscopic surgery. These principles can also be extended to other types of endoscopic surgery where other cavities are inflated to enable surgery, i.e. gastrointestinal endoscopy [15], thoracoscopic [16], colonoscopic [17], and hysteroscopic [18] procedures and open surgery [19–23]. In these situations, the tissue desiccation is of equal consequence.

A great deal of clinical research has been carried out in this area with regard to clinical outcomes such as post-operative pain, hypothermia, post-operative adhesions, recovery time and optical clarity. A summary of this research follows.

## General impact of the standard CO_2_-induced pneumoperitoneum on the body

When the standard dry and cold gas is insufflated into the warm abdomen, the gas is humidified and warmed up to reach an equilibrium of humidity and temperature, within the peritoneum. This means that the gas is warmed up until its temperature is equal to that of the peritoneum and it is humidified until it is as humid as the peritoneum. Both processes affect the patient’s condition and, more specifically, that of the peritoneum. As a consequence, the peritoneum will lose temperature and liquid to reach this equilibrium with the dry and cold gas, and this process consumes energy and consequently induces hypothermia [24, 25]. This hypothermia is mainly due to the energy spent to humidify the dry gas (577 cal to vaporise 1 g of water) rather than to the energy required to warm the cold gas (0.00003 cal to heat 1 mL of CO_2_ by 1 °C) [26]. Therefore, the pneumoperitoneum will systematically induce hypothermia [25, 27–29] that is, to a large extent, caused locally by the pneumoperitoneum-induced desiccation [30].

Other systematic effects produced by the CO_2_-induced pneumoperitoneum are the CO_2_ absorption from the abdominal cavity, causing acidosis and hypercarbia [31–35], which, if not compensated adequately for ventilation, can negatively affect the cardiovascular and respiratory functions [35, 36]. Moreover, CO_2_-induced pneumoperitoneum impairs venous return, depending on the intra-abdominal pressure [37], and decreases splanchnic perfusion with resulting oxidative stress [38]. Last but not least, CO_2_-induced pneumoperitoneum is associated with post-operative pain [39].

Other local effects produced by the CO_2_-induced pneumoperitoneum are the alteration of the peritoneal fluid [40] and induction of peritoneal acidosis [35], which may mediate suppression of peritoneal macrophage function [41]. In addition, CO_2_ alters the peritoneal microcirculation, decreasing the reactive oxygen species (ROS) scavengers [42], modulates the local immune system and the inflammatory reaction [43], and inhibits the peritoneal plasmin system, leading to peritoneal hypofibrinolysis.

As discussed above, the insufflation gas produces local and systematic effects and these side effects depend specifically on the nature of the gas: dry or humidified, cold or warm. In the following sections, the local and systematic effects will be discussed taking into account the nature of the insufflation gas.

### Impact of the insufflation gas on body temperature: hypothermia

Temperatures throughout the body are integrated by a thermoregulatory system that coordinates cold and warm defences and keeps core temperature within 0.2 °C of time-adjusted normal values [44]. General anaesthesia produces marked and dose-dependent inhibition of thermoregulatory control in a three-phase pattern. Hypothermia initially results largely from core-to-peripheral redistribution of body heat that occurs when anaesthesia inhibits tonic thermoregulation vasoconstriction. Subsequently, heat loss exceeding metabolic heat production decreases core temperature in a slow, linear fashion. Finally, a core temperature plateau results when emergence of thermoregulatory vasoconstriction decreases cutaneous heat loss and constrains metabolic heat to the core thermal compartment [45].

In addition to anaesthesia-induced hypothermia, there is another source of heat loss during laparoscopic surgery: the dry and cold insufflation gas. It had been assumed that the impact of laparoscopy would be to decrease the risk of heat loss in comparison with an open surgery [46]. During open surgery, a large area of the abdomen is exposed to air whereas during laparoscopy the abdomen is sealed. However, the abdomen is not sealed off from the laparoscopic environment. In fact, the abdomen will be in contact with the dry and cold CO_2_ and, as explained above, the gas reaches equilibrium in humidity and in temperature within the wet and warm peritoneum. Therefore, the peritoneum will lose water and temperature to reach this equilibrium, which consumes energy and consequently induces hypothermia in the patient [24, 25].

Since there are adverse clinical effects due to core temperature cooling, hypothermia should be carefully monitored [47]. Hypothermia can cause complications such as post-operative shivering, increased duration of post-anaesthetic recovery and of hospitalisation, myocardial complications, increased surgical wound infection, intra-operative blood loss, impaired platelet and immune functions, including T cell-mediated antibody production and non-specific oxidative bacterial killing by neutrophils [48].

There are numerous studies comparing the effect of different gas conditions upon hypothermia, i.e. several studies comparing the use of warm and humidified gas with the standard dry and cold gas [13, 25, 49–55], a few using dry gas comparing cold vs warm [24, 28, 56–60], two studies comparing the four gas conditions [27, 61] and two studies using standard, humidified and cold and humidified and warmed CO_2_ [62, 63].

Research into the effect of heating the dry gas to body temperature has led to mixed results. Heating the insufflation gas has been shown to reduce hypothermia [28, 56, 57], to provide no thermal benefit [24, 58, 59] and, conversely, to actually produce hypothermia [60].

When the effect of four kinds of gas (dry and cold, dry and warm, humidified and cold, humidified and warm) upon body temperature was analysed, insufflation with warm, dry gas did not prevent hypothermia; in addition, when cold CO_2_ was humidified, the decrease in core temperature was smaller than when cold, dry gas was used [27]. In a clinical trial, Davis et al. [61] analysed these four types of gas showing no differences in body temperature. However, it has to be taken into account that the study was undertaken with obese patients who have relatively less surface area from which to dissipate metabolic heat during the initial hour of surgery; therefore, the risk of intra-operative hypothermia was lower [45].

Hypothermia can be fully prevented using humidified and warm gas, as shown in animal models [25–27, 63, 64], in clinical trials [65] and as confirmed in a meta-analysis in humans [13]. However, Schlotterbeck et al. [62] have demonstrated that cold humidification of insufflating CO_2_ prevents heat loss associated with pneumoperitoneal insufflation as efficaciously as warmed humidification of the gas. The same conclusion was reached by Corona et al. [66], demonstrating that desiccation could be eliminated while maintaining the intra-peritoneal temperature between 31 and 32 °C without affecting core body temperature by insufflating humidified gas at 32 °C into the abdominal cavity. In this randomised control trial (RCT), an additional cooling to maintain temperatures of 31–32 °C in the peritoneal cavity—and avoiding desiccation—was needed and this was accomplished by nebulising 3 mL/min of water at room temperature or at 0 °C with a nozzle set. These results show that the use of humidified insufflating gas, whether heated or cold, prevents specific heat loss compared with the use of standard dry and cold insufflation gas during abdominal laparoscopy. This is consistent with the observation that much more energy is used to humidify the gas than is needed to heat it. Almost the same amount of energy is required to humidify the gas to full saturation whether the gas entering is dry at 21 °C or dry at 37 °C [46].

It is also interesting to note that the dry nature of the gas has an effect more pronounced than the gas temperature in terms of body temperature loss. Although the resulting energy loss from the body is low [67] it is the dry nature of the gas rather than the energy loss which has a more significant effect. External warming devices are effective at maintaining temperature, but cannot assist in correcting the desiccating nature of the dry gas. Whether cold or warmed, dry gas can cause cell desiccation; in fact, the warmer the gas the greater the capacity for evaporation as the gas can “hold” more water vapour. Therefore, the peritoneum will dry out faster, which potentially leads to greater adverse effects [46, 59, 68]. Only gas at body temperature and fully saturated will prevent any loss of energy from the peritoneum surface because it is physically impossible to evaporate into a fully saturated gas. As a result, the fluid layer will be maintained, minimising any energy lost from the body and, therefore, eliminating the hypothermia induced by the evaporative losses in laparoscopic surgery. In addition, several studies using humidified insufflation gas have shown that the intra-abdominal temperature has been maintained [26, 27, 52].

The surgical community is still unclear on the clinical efficacy of only heating the insufflation gas as opposed to heating and humidifying this gas. In 2002, an expert panel from The European Association for Endoscopic Surgery (EAES) published a guideline on clinical recommendations for the pneumoperitoneum during laparoscopic surgery [69]. At that time, they postulated that the clinical benefits of humidified and warmed insufflation gas were minor and contradictory. In 2006, this guideline was updated but they still postulated that “the possible and small effect of warm and humidified insufflation gas is not justified” [70]. However, research on this topic has continued from 2006, leading to supportive evidence of the benefit of using humidified and warm gas as can be seen in two meta-analysis documents published in 2008 [13, 14].

In summary, maintenance of temperature or at least a reduction in temperature loss has been demonstrated using humidified and heated gas [13, 25, 27, 65].

### Impact of the insufflation gas on pain levels

It is believed that much of the pain associated with surgery comes from the incision, however, the association between pain and wound size is not well researched. One study has demonstrated that patients with larger acute wounds reported higher pain intensity scores [71]. It is possible that when the area of the wound is larger, more nociceptors (the sensory receptors that cause the perception of pain) are activated and sensitised [72].

Through the use of laparoscopic surgery, the wound size can be reduced to only a few centimetres, considerably reducing pain and recovery time [73]. Because incision wound pain has been reduced by laparoscopic surgery, other sources of pain have become more significant and so now need to be addressed. There is evidence to suggest that the dominant source of pain and discomfort after laparoscopy is coming from the peritoneum rather than from the skin or abdominal wall [39]. One of these sources is the gas used for insufflation during laparoscopic surgery.

The cause of gas-related pain following laparoscopic surgery is multimodal. Apart from the wound size, the following points are known to be contributing factors in varying degrees: [74, 75].

#### Distension-induced neurapraxia of the phrenic nerves and pain

To allow sufficient access for the operation, insufflation pressure is usually kept at around 15 mmHg; this produces stretch-induced damage of nerves supplying the diaphragm, which possibly contributes to post-operative pain.

#### Type of insufflated gas and acidic intra-peritoneal environment and pain

The CO_2_ dissolution produces intra-abdominal acidosis which may damage the phrenic nerves and produce pain [74]. Although CO_2_ is the gas most used, other types of gas have been employed during laparoscopy, i.e. nitrous oxide (N_2_O), helium (He), argon (Ar), air and nitrogen (N_2_) [76].

Pure N_2_O was the gas preferred by gynaecologists for pneumoperitoneum in the 1970s and 1980s as N_2_O shares several advantageous properties with CO_2_, i.e. an inexpensive gas, rapid elimination, and has similar levels of diffusion and solubility. It also has anaesthetic and analgesic properties, without having the cardiopulmonary side effects of CO_2_ [77, 78]. However, N_2_O behaves like air in the presence of high concentrations of combustible gas and electrical charge and it does not suppress the risk of combustion. Due to these properties, together with two case reports of intra-operative explosion associated with its use, N_2_O has been effectively banned in therapeutic laparoscopy [79, 80]. If there is a bowel perforation and gases such as methane escape from the intestinal area and are ignited by electrosurgery, some explosion risk exists. However, this risk only exists when the concentration of N_2_O is higher than 29 % [81].

Aitola et al. [82] studied the effect of using pure N_2_O-induced pneumoperitoneum upon post-operative pain. They have demonstrated that in those patients, from whom N_2_O-induced pneumoperitoneum was used, less pain was experienced 1 and 6 h post-operatively, as well as during the next morning, in comparison with the patients for whom CO_2_-induced pneumoperitoneum was used. These results were also confirmed by a prospective single [83] and a prospective double-blind RCT [84] and by a recent meta-analysis [78]. In addition, the total amount of anaesthetic needed was lower in the N_2_O group and there were no side effects as acidosis was observed using N_2_O-induced pneumoperitoneum [82]. Moreover, the mean end-tidal CO_2_ increase was greater (despite a greater mean intra-operative increase in minute ventilation) and there was a substantial fall in the arterial pH for the patients in the CO_2_-induced pneumoperitoneum group. These phenomena were not observed in the N_2_O group [83] suggesting that CO_2_-induced acidosis may be involved in peritoneal irritation resulting in pain. In an RCT in deep endometriosis surgery [85], full-conditioning (86 % CO_2_ 10 % N_2_O 4 % O_2_ for the pneumoperitoneum, cooling of the peritoneal cavity to 32 °C, humidification), heparinised rinsing solution and 5 mg of dexamethasone showed reduced post-operative pain, together with a lower CO_2_ resorption and less inflammation (lower C-reactive protein concentrations), in comparison to that of the CO_2_ pneumoperitoneum group. In this trial, it was postulated that the pain was mainly reduced by adding a small amount of N_2_O to the pneumoperitoneum, however, the effect of dexamethasone and local cooling upon pain cannot be excluded.

It is believed that helium-based pneumoperitoneum induces less post-operative pain due to its properties of being an inert gas, which has a more limited effect on intra-abdominal pH and metabolism in comparison to the use of CO_2_ [86]. However, when helium-induced pneumoperitoneum was used, patients reported similar pain scores to those under CO_2_-induced pneumoperitoneum [12, 78, 86]. Fewer cardiopulmonary changes were observed with helium-induced pneumoperitoneum than CO_2_-induced pneumoperitoneum and there were no significant differences in cardiopulmonary complications and surgical morbidity [78]. Interestingly, O’Boyle et al. [86] studied the effect of helium and CO_2_-induced pneumoperitoneum together with the effect of saline lavage upon pain. Less pain was found in the group undergoing saline peritoneal lavage, demonstrating the importance of keeping the abdominal cavity wet.

In summary, there is an association between CO_2_-induced intra-peritoneal acidosis and pain. This acidosis might be avoided using helium or N_2_O insufflation gas. However, more clinical studies are needed to confirm the validity and safeness of these gases during laparoscopic surgery. In spite of the CO_2_-induced acidosis, CO_2_ is the most common gas used and nothing is known about the other gases, i.e. argon, nitrogen and air, and their relation with post-operative pain.

#### Residual intra-abdominal gas after laparoscopy and pain

Shoulder tip pain can be understood by considering the effect of residual CO_2_ gas. After surgery, a volume of insufflation gas remains in the peritoneal cavity for up to 3 days [87] and tends to collect at the top of the cavity under the diaphragm [88]. These gas bubbles are thought to irritate the diaphragm and the phrenic nerve, thus leading to subcostal pain. Because the nerves of the shoulder and the diaphragm exit the spine in the same bundle, irritation in one area can cause the brain to sense pain at both sites. Clinical studies showed that the severity and duration of post-operative pain was proportional to the amount of CO_2_ that remained in the pneumoperitoneum after laparoscopic surgery [88, 89].

Benefits from removal of this gas can be seen using an intra-peritoneal drain, which showed a decrease in the frequency of shoulder pain and a reduction in the post-operative analgesia requirements after laparoscopy [90, 91]. However, a study showed that post-operative pain was significantly increased in patients who had a drain in position compared with those in the non-drained group [92]. In addition, drains can produce some complications, such as an increase in wound infection rates and a delayed hospital discharge [93].

A quantification of the removal rate of gas bubbles has been undertaken by Glew et al. [94] and it was shown that gas bubbles can be removed significantly faster using humidified gas. Humidifying the gas can assist in the removal of residual CO_2_ due to its high solubility. To migrate through the tissue into the bloodstream, the gas needs to be dissolved in a fluid. The dry nature of the gas causes evaporation of the serous fluid. The remaining fluid is viscous [40], reducing the dissolving rate of the CO_2_; thus, the gas remains in the peritoneal cavity longer. It has been shown that using humidified insufflation gas, the serous fluid in the peritoneum will remain moist [40] facilitating the dissolving of CO_2_ and absorption out of the peritoneum faster, therefore reducing post-operative pain [88].

It is also known that there is a “suction effect” between the diaphragm and the liver and this is interrupted following surgery. This effect can be explained by means of an easy example, i.e. when a piece of paper is placed over a glass of water and the glass is inverted, the paper is held in place. Much of the weight of the liver is carried in a similar fashion, so the load of the liver is distributed across much of the upper peritoneal cavity. It has been proposed that the gas remaining in the peritoneum after insufflation interrupts this suction facility shifting more of the load to the mechanical fastenings between the liver and the diaphragm [75, 87, 95]. The resulting localised strain is then a source of irritation to the diaphragm and a likely cause of subcostal and referred shoulder tip pain. Support to this “suction effect” hypothesis is given by the location of the liver within the right hypochondriac region and by the observation that pain is often more severe in the right shoulder tip and the right subcostal areas. Humidification of insufflation gas helps return suction support sooner by preventing evaporation of the fluid on peritoneal and liver surfaces, and by faster removal of the gas pocket [94] causing the loss of suction. Some studies have shown partial success in correcting for the suction effect by spraying the peritoneal cavity with saline [87].

In summary, the residual intra-abdominal gas remaining after the surgery produces post-operative pain and an interruption to the suction effect; both can be improved using humidified gas.

#### Temperature and humidification of the insufflated gas and pain

The effect of the insufflation gas temperature upon post-operative pain is controversial [59, 75, 96, 97]. Korell et al. [97] demonstrated that the use of dry and warm gas reduced pain levels in a prospective randomised study. In another clinical trial, the effect of three gas conditions (humidified and heated; dry and heated; standard dry and cold gas) upon post-operative pain was investigated and no significant difference in intra-operative and post-operative analgesic requirements or post-operative pain score was found [96] There was even a tendency (although not significant) toward less pain and higher post-operative satisfaction among patients in the control group (standard dry and cold gas). However, a criticism to this study is the small sample size (*n* = 53). On the other hand, another prospective, controlled, randomised, double-blind study demonstrated that using humidified-warm gas for laparoscopic gastric banding reduces shoulder pain, and decreases pain medication requirements for up to 10 days post-operatively in comparison with gas conditions used for the other groups. In addition, dry-heated gas may cause additional complications since this increases pain medication use and pain intensity [98]. In this study, the sample size was bigger (*n* = 113).

In another study, it was demonstrated that patients receiving heated dry gas had more early post-operative pain than those in the control group using room temperature gas, suggesting that heated gas has no benefit in terms of pain reduction [68]. The authors suggested that the drying effect of the gas could be the cause. Consistent with this, the shoulder tip and subcostal pains were more intense after using warm gas during laparoscopy [59].

A possible explanation to the results obtained by the last three studies can be due to the characteristics of a dry gas. It is known that the capacity of a gas to retain water depends on its temperature: the higher the temperature, the more water a gas can hold. Therefore, when a dry gas enters the abdominal cavity, desiccation will inevitably occur [30] and it will increase at higher temperatures. In addition, the peritoneum has a large surface with a thin serous fluid layer which facilitates humidification of the pneumoperitoneum gas. As a result, a heated gas will produce more desiccation in the abdominal cavity than does a room temperature gas and this peritoneal damage may cause more pain.

With regard to the use of humidified gas, many clinical studies have demonstrated that patients receiving humidified and heated insufflation gas experienced less post-operative pain. This can be seen in a variety of procedures: laparoscopic cholecystectomy [51], several conscious [99, 100] and unconscious gynaecological procedures [50], several thoracoscopic procedures [16], gastric bypass [101], and a further study with Nissen fundoplication showed a beneficial trend but due to the low number of patients did not reach statistical significance [52]. Moreover, two meta-analyses have been published showing that patients in the humidified and warm insufflation gas group experienced a significant reduction in pain score after surgery, and in their analgesic requirements than did those in the control group which had standard cold and dry CO_2_ [13, 14].

For shorter surgeries, such as acute laparoscopic appendectomy, it was found that using humidified gas does not impart any clinical benefit on post-operative pain, on intra-operative core temperature and on post-operative recovery parameters in paediatric patients [102].

#### Tissue drying, peritoneal damage and post-operative pain

The exact relation between the level of tissue damage and post-operative pain is difficult to determine. Several animal studies have shown that dry and cold gas is deleterious for the peritoneum, i.e. it destroys the microvilli, causes the mesothelial cells to retract and bulge and exposes the basal lamina [27, 64, 65, 103–106]. Interestingly, these alterations depend on the duration of gas insufflation [107] and the insufflation pressure and type of gas [108]. When humidified and heated CO_2_ is used, fewer changes to the peritoneal layer were observed in comparison to using dry and cold gas in rats [64, 103] and in pigs [65]. However, two studies did not observe any improvement of the peritoneal surface after humidified CO_2_ insufflation [27, 94]. In the Hazebroek et al. study, anaesthesia was induced and maintained with repeated intra-peritoneal injections of pentobarbital sodium. Thus, some of the observed damage could be attributed to the direct effect of pentobarbital on the peritoneal surface. In the Glew et al. study, the samples were analysed by light microscopy, a technique less sensitive than the scanning electronic microscopy.

There is only one clinical trial in which the four types of gases were used in patients and both peritoneal morphology and pain scores were evaluated [61]. In this trial, peritoneal samples were taken at the beginning and at the end of the surgery, and no difference in either the pain scores or in the peritoneal morphology was found. This led the authors to postulate that “heating or humidifying of CO_2_ is not justified for patients undergoing laparoscopic bariatric surgery”. However, this trial has several limitations, i.e. the sample size, power of the study and data collection, which can lead to erroneous conclusions [109].

In summary, although the exact relation between tissue damage and post-operative pain is difficult to determine, it is clear that dry and cold gas damages the peritoneum and that both pain and tissue damage are avoided using humidified gas.

#### Volume of the insufflated gas and pain

Pain increases with the gas consumption, a fact which led to the belief that the pain is caused by a physical effect [110]. For instance, it was demonstrated that the volume of the CO_2_ insufflated is an important factor in the cause of pain since post-operative pain levels increased with a high insufflation rate [111] but not with the duration of the surgery [112]. This indicates that the level of desiccation may be the contributing factor.

#### Pressure used to induce the pneumoperitoneum and pain

It was demonstrated that insufflation pressure significantly increased the post-operative pain associated with laparoscopic cholecystectomy [113, 114].

In summary, pain during surgery can be produced by several sources: the distension of the phrenic nerves, the type of insufflated gas and acidic intra-peritoneal environment, the residual intra-abdominal gas, the temperature and humidification of the insufflated gas, the tissue drying and the volume and pressure of the insufflated gas. Many of the factors causing post-operative pain listed at the beginning of this section can be reduced by humidifying and heating the gas.

### Impact of the insufflation gas on cellular integrity and tissue damage

As explained in the section above regarding “Tissue drying, peritoneal damage and post-operative pain”, the thin layer of mesothelial cells covering peritoneal surfaces is partly or completely damaged when dry gas is used, i.e. microvilli are destroyed, cells become retracted and bulged, the intercellular clefts increase in size and the basal lamina is exposed [27, 64, 65, 104, 105, 107, 115, 116], and this can be reduced using humidified and warm gas [64, 65, 103, 116].

This damage can be explained by the fact that when the insufflation gas exits the cannulae into the peritoneum and the gas travels as a jet stream until it reaches the wall of the peritoneum or an organ, where it is deflected [117]. Exit velocities approached 30 m/s when the hydraulic diameter was 3 mm and differential pressure was 12 mmHg. If the gas is dry, fluid is evaporated off the tissue in the localised region where the gas impinges; this evaporation causes a severe cooling and tissue drying which results in tissue damage and pain [30, 118].

This mode of tissue damage is not possible if the gas is already saturated with water vapour. When the gas is holding water at 100 % relative humidity (RH) at the same temperature as the surrounding tissue (around 37 °C), no further water can be absorbed. Evaporation from the serous fluid will not occur and cooling and cell desiccation will not take place [30]. Evaporation of serous fluid from the peritoneal surface due to the dry gas also causes less severe but much wider spread tissue damage in the rest of the peritoneum.

Following the peritoneal trauma due to the desiccating nature of the dry gas, an inflammatory reaction is produced, as demonstrated in several animal models. Two hours after a laparoscopy was performed with dry and cold CO_2_, an inflammatory cell infiltration in the parietal and visceral peritoneum was observed [119]. Volz et al. [104] showed that, 12 h after the laparoscopy, peritoneal macrophages and lymphocytes filled all gaps, recovering the basal lamina, as confirmed by Davey et al. [116]. These results in animal models were confirmed in humans by Liu et al. [107], demonstrating that 2 h after dry CO_2_ insufflation a small amount of lymphocytes and macrophages were found in the intercellular clefts.

Moreover, the degree of inflammation will also depend on the type of gas and the insufflation pressure used during laparoscopy. Paparella et al. [108, 119] demonstrated that air-induced pneumoperitoneum produces more inflammation than CO_2_-induced pneumoperitoneum by evaluating the following features of the peritoneal surface: congestion, haemorrhage, oedema, and inflammatory cells and their location of the lamina propria and submucosal, and mesothelial cells looking for hyperplasia, metaplasia and hypertrophy. They also demonstrated that at higher insufflation pressure the inflammation is greater [108]. The trauma associated with the higher insufflation pressure was also demonstrated by the group of Matsuzaki et al. [120, 121] that showed that a low intra-peritoneal pressure (8 mmHg) would be better than the standard intra-peritoneal pressure (12 mmHg) to minimise the adverse impact on the surgical peritoneal environment, and on the peritoneal fibrinolytic system during a CO_2_ pneumoperitoneum [122].

When dry-heated CO_2_ was insufflated, some animals showed little or no alteration of the mesothelial layer, while others had a mild inflammatory response and mesothelial cells were rounded and showed crenation on the exposed surface; when insufflation was performed with heated and humidified CO_2_, specimens showed little change when compared with those within the control group [116]. Humidified and heated gas reduces the inflammatory response as seen in the reduction of tumour necrosis factor alpha (TNF-α) [94]. In addition, another study has shown that the use of humidified gas diminishes the increased lymphocytes which occurred during laparoscopy [103]. This shows that less trauma occurs in the peritoneum with humidified gas.

Interestingly, Corona et al. [123] have demonstrated that manipulation of the upper bowel during laparoscopy induced with CO_2_ increased acute inflammation at the abdominal cavity in comparison to those in the control group without using manipulation. This shows the importance of good surgical practice.

### Impact of the insufflation gas on the recovery time

The time taken for a patient to recover from surgery is an important issue. Any time saved at each point of recovery also contributes to a reduction in the cost of treatment and the quality of life of the patient.

#### Recovery room stay

Directly after surgery, a patient is transferred to the recovery unit until they are sufficiently stable and conscious to be transferred to a normal ward or to return home. A patient’s fitness to be discharged from recovery is based on factors such as respiratory and circulatory baselines, body temperature and levels of consciousness and pain. In the case of a healthy patient, hypothermia and pain are the factors most likely to require a patient to stay longer in the recovery ward. Reduced hypothermia and decreased pain levels (by faster removal of gas and less trauma to the peritoneal tissue) mean that a patient will require less time to recover.

Two studies have shown that patients who received humidified insufflation gas during surgery spent significantly less time in the recovery unit [50, 98]. It was shown that 89 % of patients with humidified gas stayed in the recovery unit less than 1 h, but only 33 % of patients with cold dry gas stayed under 1 h [50]. This potentially reduces “bottlenecks” in the recovery department and costs. However, several studies did not find any significant differences in the recovery room stay using humidified gas [55, 61, 124]. Manwaring et al. [124] suggested that external warming blankets may be more effective in maintaining intra-operative normothermia than the use of heated humidified gas.

#### Hospital stay

Some studies have shown that the use of humidified insufflation gas leads to a shorter hospital stay when compared to cold dry insufflation gas [54]. This can translate to significant cost benefits for the hospital. However, several studies did not show any benefit in the length of hospitalisation when humidified gas is used compared with dry and cold gas usage [16, 52, 53, 55, 61] information confirmed by a meta-analysis [13].

#### Return to normal activities

After a patient is discharged from hospital, there is a recovery period before they are able to return to normal daily activities and work. It has been shown that if humidified and warm gas is used during laparoscopic cholecystectomy procedures, the recovery time to recommence normal activities can be reduced and it was halved compared to the use of standard cold and dry CO_2_ insufflation gas [51]. However, two studies did not show any significant differences using humidified gas [53, 125].

In summary, although it is clear that humidified and warm gas prevents hypothermia and pain after surgery, results related to the patient recovery are still controversial. Of course the recovery time depends on several factors, i.e. patient characteristics, surgeon skills, type and duration of the surgery, and makes this topic difficult to fully evaluate.

### Impact of the insufflation gas on post-operative adhesions

#### Definition, aetiology and incidence of intra-peritoneal adhesions

Adhesions are abnormal fibrous connections between surfaces within body cavities. Many different insults, such as infections, surgery, chemical irritation, endometriosis and dry gas, can disrupt the peritoneum, produce inflammation and develop adhesions [126]. Such adhesions can be classified, according to the aetiology, in congenital or acquired, either post-inflammatory or post-operatively [127]. Abdominal surgery is the most common cause of adhesions; the incidence ranges from 63 to 97 % [127–129]. Some 10 % of patients without having had previous surgery have adhesions (92 % post-inflammatory and 8 % congenital). In contrast, adhesions are found in 93 % of patients with at least one previous surgery (98 % post-operative, 1 % post-inflammatory and 1 % congenital) [128].

#### Clinical significance of intra-peritoneal adhesions

Adhesions are the major cause of intestinal obstruction [130, 131], of female infertility [132, 133], chronic pain, and difficulties at the time of re-operation. The burden of post-operative adhesions is best illustrated by the study showing that 5.7 % of all readmissions of patients undergoing open abdominal or pelvic surgery were classified as being directly related to adhesions, and 3.8 % of the patients were managed operatively [127]. In addition, 34.6 % of the patients who underwent open abdominal or pelvic surgery were readmitted 2.1 times over 10 years for a disorder directly or possibly related to adhesions and 22.1 % of all outcome readmissions occurred in the first year after initial surgery. Last but not least, the financial consequences of the adhesions are enormous, i.e. adhesiolysis hospitalisations during 1988 in USA accounted for US$1180 million in healthcare expenditures, with US$925 million going towards in-hospital expenses and US$255 million for surgeon fees [134].

#### Pneumoperitoneum as a cofactor in adhesion formation

It has been claimed that laparoscopy is less adhesiogenic than laparotomy but the data are inconclusive. Animal studies indicate that laparoscopy could induce less adhesion formation [135–138], whereas other studies fail to show differences [139–141]. In humans, laparoscopy could induce less de novo adhesion formation [142, 143] but for adhesion reformation, this also remains controversial [142–145].

To interpret this data, it is important to highlight the differences between laparoscopy and laparotomy in terms of the direct trauma induced by the surgery itself and the indirect trauma that might be induced by the peritoneal environment. If performed adequately by well-trained surgeons, laparoscopy should induce less direct surgical trauma because of gentle tissue handling, meticulous haemostasis, constant irrigation, the use of microsurgical instruments and the smaller operative field, which may reduce the risk of adhesion formation. On the other hand, laparoscopy and laparotomy are performed in different gas environments: CO_2_ for the former and air for the latter. Indeed, as explained above, the peritoneum is not designed to cope with the variable conditions of the pneumoperitoneum. Thus, the type of gas used to induce the pneumoperitoneum, the nature of the gas (temperature and humidity) and the pressure can all cause tissue damage which results in an increase in adhesion formation.

It has been postulated in studies of mice and rabbits that CO_2_-induced pneumoperitoneum is a cofactor in adhesion formation [146–148]. In these models, standardised lesions were performed during laparoscopy and the effect of different factors with regard to the pneumoperitoneum (duration, gas type, pressure, humidified or dry gas) has been studied. It was demonstrated that adhesions increased with duration of pneumoperitoneum and with insufflation pressure [146, 148–150]. Furthermore, a reduction of adhesions has been observed when a small amount of oxygen (3–4 %) is added to both CO_2_ and helium-induced pneumoperitoneum in rabbits [149, 150] and mice [146, 148]. Therefore, it was assumed that the CO_2_ pneumoperitoneum-enhanced adhesions could be mediated by mesothelial hypoxia. The beneficial effect of the addition of 3–4 % oxygen can be explained by the fact that the mesothelial cells would be in a more physiologic (normoxic) environment [151, 152]. This hypothesis is supported by the observation that during CO_2_ or helium-induced pneumoperitoneum, the partial pressure of oxygen in the abdominal wall was reduced [153]. Moreover, this is consistent with the result of Matsuzaki et al. [154] showing that a perioperative oxygen supplementation at the ventilation reduced post-operative adhesion formation through increasing the peritoneal tissue oxygen tension.

In addition, the relation between CO_2_ pneumoperitoneum-induced acidosis/hypercarbia and adhesion formation has also been addressed in a laparoscopic mouse model in which animals with endotracheal intubation were mechanically ventilated with different patterns [32]. Adhesion formation was higher in animals poorly ventilated and decreased with higher ventilation rates. In comparison with animals that underwent anaesthesia only, the CO_2_-induced pneumoperitoneum increases the pCO_2_ and decreases the pH, as has been reported in animal models [31, 34, 155] and humans [36]. These effects were more pronounced in poorly ventilated mice and counteracted by appropriate ventilation (i.e. higher ventilation rates). These data demonstrate an association between CO_2_ pneumoperitoneum-induced acidosis/hypercarbia and adhesion formation. However, the mechanism whereby this acidosis/hypercarbia becomes a cofactor in adhesion formation remains unclear. Obviously a trauma produced by the acidosis/hypercarbia upon mesothelial cells and molecules involved in adhesion formation cannot be excluded. Indeed, acidosis affects lymphocyte and macrophage functions altering cellular and humoral immune function [156], acidosis increases the release and production of plasminogen activator inhibitor 1 (PAI-1) by the mesothelial cells [157] and up-regulates vascular endothelial growth factor (VEGF) expression independently from hypoxia [158], which has been reported to be involved in adhesion formation [159–163].

Moreover, the effect of other gases has been studied with regard to adhesions. It was demonstrated that helium-induced pneumoperitoneum caused the same quantity of adhesions to that of CO_2_-induced pneumoperitoneum [146] and that adding 5–10 % of N_2_O to the CO_2_-induced pneumoperitoneum significantly reduced post-operative adhesions [164]. The exact effect of N_2_O upon post-operative adhesions cannot be explained by the current knowledge. It was hypothesised that N_2_O may be reducing the acute inflammation at the peritoneal cavity in comparison with that evident in CO_2_-induced pneumoperitoneum.

It has been claimed that the desiccation caused by the standard dry and cold CO_2_ pneumoperitoneum will favour the development of post-operative adhesions. In vitro studies confirm that the degree of desiccation depends on the flow rate of the gas through the humidified surface. Indeed, when dry and cold CO_2_ circulates through water-filled flasks, the water lost depends on the flow rate; the higher the flow, the more desiccation is observed [148]. The effect of dry CO_2_ with different insufflation pressures and flow rates through the abdominal cavity upon adhesion formation was evaluated, showing that adhesion formation increased with higher insufflation pressures and with higher flow rates [26, 148, 150]. Furthermore, this desiccation-induced adhesion formation was reduced using warm and humidified gas in mice [26] and in rats [64]. Therefore, the key role of desiccation in the pathogenesis of the adhesion formation is evident. The hypothesis of desiccation as a driving mechanism in adhesion formation is supported by the data demonstrating that the dry and cold CO_2_-induced pneumoperitoneum alters the morphology of the mesothelium as explained in detail previously [27, 64, 65, 104, 105], which can favour the development of post-operative adhesions.

The effect of using humidified insufflation gas to reduce adhesions is clear. Therefore, the effect of using humidified gas at different temperatures was also studied showing that hypothermia reduced adhesion formation in mice [26, 165]. Consistent with these results, animal data demonstrated that peritoneal infusion with cold saline at 4 °C decreased post-operative adhesions [166], whereas irrigation with saline at warmer than body temperature increased post-operative adhesions [167]. Recent experiments confirmed that peritoneal infusion with cold saline at 4 °C decreased post-operative adhesions and same results were obtained using cold saline at 10 and 15 °C [168]. Several mechanisms might be involved in this beneficial effect of hypothermia. Adhesion formation might be reduced by hypothermia through protecting tissues and cells from the pneumoperitoneum-induced hypoxia, since cell oxygen consumption decreases with temperature. Indeed, hypothermia decreases the global cerebral metabolic rate during ischaemia, slowing the breakdown of glucose, phosphocreatine and adenosine triphosphate (ATP) and the formation of lactate and inorganic phosphate [169]. In addition, hypothermia reduces the production of ROS during reperfusion [170–172], improves the recovery of energetic parameters during reperfusion [169], and suppresses the inflammatory response thus decreasing the infiltration of polymorphonuclear (PMN) cells and the production of TNF-α, interleukin 1β (IL1β) and macrophage inflammatory protein-2 [173, 174]. In the recent article of Lin et al. [168], intra-peritoneal cold infusion at 4, 10 and 15 °C has shown a decrease of post-operative adhesions together with a decrease of the levels of TNF-α and interleukin 6 (IL6) compared with those in the group without saline infusion.

These results were further translated to clinical trials showing that it is possible to insufflate humidified and cold gas (32 °C, 100 % RH) reducing the abdominal temperature locally but without affecting the core body temperature [66]. In an RCT in deep endometriosis surgery [85], post-operative adhesions were completely prevented in 12 out of 16 women using full-conditioning (86 % CO_2_ + 10 % N_2_O + 4 % O_2_ for the pneumoperitoneum, humidification and cooling of the peritoneal cavity to 32 °C), heparinised rinsing solution and 5 mg of dexamethasone together with a barrier, whereas in the control group with humidified CO_2_ at 37 °C (*n* = 11) all women had severe adhesions. Also the area, density and severity of adhesions were significantly less. In the control group, area, density and severity of adhesions were strongly interrelated suggesting a common enhancing factor. In the full-conditioning group, CO_2_ resorption, post-operative pain and CRP concentrations were lower while clinical recovery was faster and time to first flatus shorter.

In conclusion, animal data indicate that the standard dry and cold CO_2_-induced pneumoperitoneum is a cofactor in adhesion formation because it induces hypoxia, acidosis and desiccation. Peritoneal adhesions can be reduced to a large extent with adequate pneumoperitoneum conditioning i.e. by adding 3–4 % O_2_ to avoid hypoxia, adding 5–10 % N_2_O to reduce (possibly) inflammation, humidifying the insufflation gas to avoid desiccation, and by slightly cooling the insufflation gas to reduce inflammation. The relevance of all these strategies for peritoneal environment conditioning, together with the application of a barrier and dexamethasone, was translated to humans to reduce adhesion formation [85]. This trial was a “first proof of concept” trial of adhesion formation in a limited number of patients and these results should be confirmed in other RCTs.

## Others: impact of the insufflation gas on the optical clarity

When a cold object such as a laparoscope is introduced into a warm humid environment such as the abdomen, moisture in the environment condenses on the object, in this case the rigid laparoscope lens. If cool insufflation gas is continuously passing over the laparoscope during the course of an operation, it remains colder than the surrounding environment and it continues to fog up. Therefore, this imbalance between the temperature of the front lens of the laparoscope and that of the abdominal cavity will produce the laparoscopic lens fogging [175]. Lens fogging is a problem during laparoscopic surgery because a poor picture means that surgery must be stopped until the camera is cleaned and the picture stored, and may, therefore, contribute to surgical errors. Although this stoppage is rarely dangerous, it is frustrating for the surgeon and increases surgery time.

It is common practice to use anti-fogging solutions or devices such as a laparoscope warmer to prevent fogging. However, these methods fail to maintain the warm temperature at the front lens of the scope. Irrigation channels allow rinsing of the lens, but leave a residual liquid film layer which distorts the view [176]. Furthermore, when irrigation fluid is sprayed on the lens, if the fluid temperature is less than 37 °C, it will cool the lens and thus contribute to subsequent fogging.

If the insufflation gas has been heated, the laparoscope will warm up and is less prone to fogging [176]. As long as humidified gas passing the laparoscope is slightly warmer than the dew point of the gas, there should be no condensation and hence no fogging on the lens. However, some studies demonstrated that lens fogging rates do not differ using humidified gas [13, 52, 53, 55]. Conversely, the median camera fogging score was significantly worse in the group in which humidified gas was used in a multi-centre, double-blinded, randomised controlled study during laparoscopic colonic surgery [125].

In summary, although the aetiology of laparoscopic lens fogging is well understood, i.e. the temperature and humidity differences, the methods to reduce its occurrence lack significant data. Of those methods that are often espoused, most are not supported in the literature, such as warmed and humidified insufflation gas, or simply lack data, such as anti-fogging solutions [175].

## Conclusion

The peritoneum, one of the largest organs in humans, has a very important function in the abdominal cavity, i.e. diminishes the friction, walls off infections and enables the secretion of cytokines. It is a very delicate layer highly susceptible to damage. Of course, it is not designed to cope with variable conditions such as being in contact with dry and cold air or CO_2_ during open and laparoscopic surgery, respectively. In this review, the effect of insufflating into the abdominal cavity with dry and cold CO_2_ was revised in detail.

Insufflating dry and cold CO_2_ into the abdominal cavity causes peritoneal damage, post-operative pain, hypothermia and post-operative adhesion formation. After the peritoneal trauma due to the desiccating nature of the dry gas, an inflammatory reaction is produced and peritoneal macrophages and lymphocytes will fill the gaps to recover the basal lamina. On the other hand, when humidified and heated CO_2_ is used, the peritoneal layer showed little change. Humidified and heated gas reduces the inflammatory response demonstrating that less trauma is incurred to the peritoneum. In addition, it has been clearly confirmed by clinical trials that warm and humidified gas prevents pain after surgery [13, 14].

Results related to patient recovery are still controversial. The recovery time is difficult to evaluate since it depends on several factors such as patient characteristics, surgeon skills, type of surgery and duration of the surgery.

With regard to hypothermia due to desiccation, it can be fully prevented using humidified and warm gas, as shown in animal models and in clinical trials [13]. A few studies using humidified and cold CO_2_ as insufflation gas have shown that the prevention of heat loss and desiccation associated with pneumoperitoneal insufflation are as effective as the use of warmed and humidified gas in animal models [62, 177] and in humans [66, 85]. This is consistent with the observation that much more energy is used to humidify a gas than to heat it up. Then, why use humidified and cold gas when the abdominal cavity’s normal temperature is 37 °C? The use of humidified and cold gas would produce locally hypothermia and may have many protecting effects against trauma. First, hypothermia suppresses the inflammatory response, decreasing the infiltration of PMN cells, and the production of TNF-α, IL-1β and macrophage inflammatory protein-2 [168, 173, 174]. Second, hypothermia would directly protect tissues from the pneumoperitoneum-induced hypoxia, since oxygen consumption decreases with temperature. Third, hypothermia reduces the production of ROS during reperfusion in several organs [170–172, 178–180]. Fourth, hypothermia improves recovery of energetic parameters during reperfusion [169]. Last and not least, hypothermia decreases post-operative adhesions in animal models [26, 165, 166, 168] and this can easily be translated to humans by inducing a local hypothermia of 32 °C [66].

In summary, using humidified and warm insufflation gas now offers a significant clinical benefit to the patient, creating a more physiologic peritoneal environment and reducing the post-operative pain and hypothermia. Moreover, although humidified and warm gas reduces post-operative adhesions in animal models, humidified gas at 32 °C decreased it even more. It is clear that humidified gas should be used during laparoscopic surgery; however, a question remains unanswered: to achieve even greater clinical benefit to the patient, at what temperature should the humidified gas be when insufflated into the abdomen? More clinical trials should be performed to resolve this query.
